# The CRISPR/Cas Genome-Editing Tool: Application in Improvement of Crops

**DOI:** 10.3389/fpls.2016.00506

**Published:** 2016-04-19

**Authors:** Surender Khatodia, Kirti Bhatotia, Nishat Passricha, S. M. P. Khurana, Narendra Tuteja

**Affiliations:** ^1^Amity Institute of Biotechnology, Amity University HaryanaGurgaon, India; ^2^Plant Molecular Biology Group, International Centre for Genetic Engineering and Biotechnology New Delhi, India; ^3^Amity Institute of Microbial Technology, Amity UniversityNoida, India

**Keywords:** CRISPR, Cas9/sgRNA, plant genome editing, GE crops, dCas9, RGENs, NPBTs

## Abstract

The Clustered Regularly Interspaced Short Palindromic Repeats associated Cas9/sgRNA system is a novel targeted genome-editing technique derived from bacterial immune system. It is an inexpensive, easy, most user friendly and rapidly adopted genome editing tool transforming to revolutionary paradigm. This technique enables precise genomic modifications in many different organisms and tissues. Cas9 protein is an RNA guided endonuclease utilized for creating targeted double-stranded breaks with only a short RNA sequence to confer recognition of the target in animals and plants. Development of genetically edited (GE) crops similar to those developed by conventional or mutation breeding using this potential technique makes it a promising and extremely versatile tool for providing sustainable productive agriculture for better feeding of rapidly growing population in a changing climate. The emerging areas of research for the genome editing in plants include interrogating gene function, rewiring the regulatory signaling networks and sgRNA library for high-throughput loss-of-function screening. In this review, we have described the broad applicability of the Cas9 nuclease mediated targeted plant genome editing for development of designer crops. The regulatory uncertainty and social acceptance of plant breeding by Cas9 genome editing have also been described. With this powerful and innovative technique the designer GE non-GM plants could further advance climate resilient and sustainable agriculture in the future and maximizing yield by combating abiotic and biotic stresses.

## CRISPR/Cas Technology – An Overview

**FIGURE 1:**
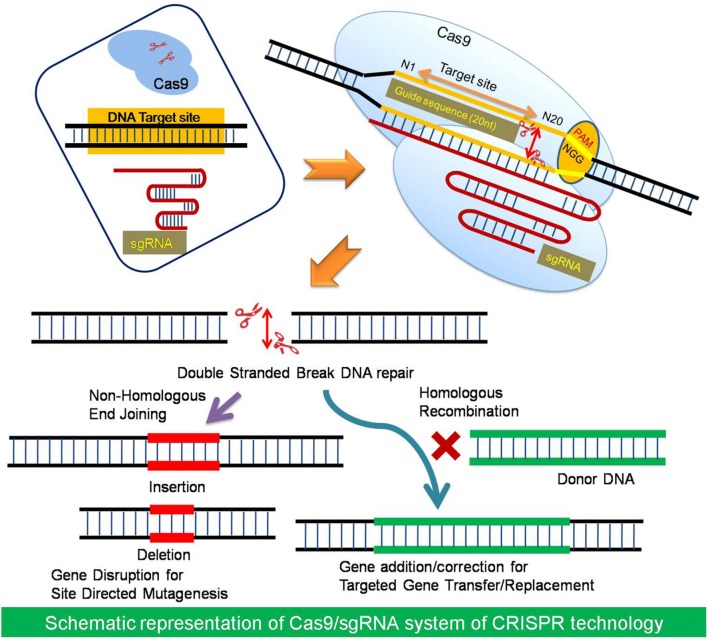
**The basic strategy of Cas9/sgRNA system.** The Cas9 is a RNA guided endonuclease consists of two nuclease domains namely HNH and RuvC. The target specificity of Cas9 depends upon the guide sequence (20 nt) short guide RNA (sgRNA). The target sites must lay immediately 5′of a PAM (Protospacer Adjacent Motif) sequence of the form N20-NGG (or N20-NAG). The Cas9 nuclease induces double stranded breaks (DSB) at the target site which can be repaired either by Non- Homologous End Joining method or Homologous Recombination by cellular system which results in gene disruption by indels or gene addition/correction, respectively.

Clustered regularly interspaced short palindromic repeat/Cas system was discovered in bacteria as an adaptive immune system which helps the bacteria in protecting itself against invading foreign DNA, such as that of a bacteriophage. This system comprises of CRISPR loci in the genome and a Cas9 protein. CRISPR, i.e., Clustered Regularly Interspaced Short Palindromic Repeats (CRISPRs) – is a genomic locus of tandem direct repeat sequences and protospacers, the spaces in between repeat sequences, both of which are derived from the invading elements (Kim and Kim, 2014). The CRISPR loci contains a combination of Cas9 genes; sequences for non-coding RNA elements called CRISPR RNA (crRNA) and sequences for small trans-encoded CRISPR RNA, i.e., trans-activating crRNA (tracrRNA). The two RNA sequences crRNA and tracrRNA forms a complex known as guide RNA, which determines the specificity of the cleavage of the target sequence in the nucleic acid along with the Protosapcer Adjacent Motif (PAM), a 5′-NGG sequence (Barrangou, 2013; Jinek et al., 2013). The cleavage of the double stranded target DNA occurs within the limits of protospacer region. The Cas9 protein is an endonuclease associated with CRISPR loci, which is responsible for the double-stranded breaks (DSBs) at the site, when targeted by a guide RNA (Cong et al., 2013; Mali et al., 2013). This revealing of molecular mechanism of the CRISPR/Cas system in 2012 opened up its vast area of applications as a promising component of genome editing termed as RNA-guided engineered nucleases (RGENs), which were used as sequence specific nucleases for precise genetic modifications (Doudna and Charpentier, 2014; Fichtner et al., 2014; Liang et al., 2015). RGENs are developed as programmable nucleases composed of two components, which must be expressed in cells to perform genome editing; the Cas9 nuclease and an engineered single guide RNA (sgRNA). The sgRNA has 20 nucleotides at the 5′ end that directs Cas9 to the complementary target site. Any DNA sequence of the form N20-NGG can be targeted by altering the first 20 nucleotides of the gRNA for novel genome editing applications (Sander and Joung, 2014) (**Figure 1**).

The targeted genome editing is utilized for the generation of desired endogenous modifications like gene disruption, addition, or correction at one or more specific genome site by introduction of nuclease mediated DNA-break using customized engineered nucleases (Kim and Kim, 2014). After DSB the cellular recombination repair mechanism can do the desired modification in a broad range of organisms and cell types. The elucidation of the CRISPR-Cas9 mechanism have resulted in many fundamental discoveries in biology (Doudna and Charpentier, 2014). CRISPR/Cas genome editing as a fledgling technology has reinvented the genetic and molecular biology research due to its simplicity and ease of design. There are many Cas9 and gRNA variants available, which could be utilized for further novel applications particularly in the field of plant biotechnology.

## The Cas9 Nuclease Variants

The Cas9 endonuclease consists of two different domains, which includes a large globular recognition (REC) specific functional domain, connected to a smaller nuclease (NUC) domain. The NUC domain further accommodates two nuclease sites, RuvC and HNH, and also a PAM-interacting site (Doudna and Charpentier, 2014; Jinek et al., 2014). Cas9 protein is activated upon the loading of guide RNA, which further undergoes a conformational rearrangement to form a central channel for RNA-DNA heteroduplex binding and canonical PAM motifs recognition (Anders et al., 2014). The mechanism of Cas9 nuclease action was recently elucidated by crystal structure studies in a complex with partially duplexed target DNA containing PAM motif and sgRNA. This provides an insight into how Cas9 may be engineered to create variants with novel PAM specificities (Belhaj et al., 2013). There are many Cas9 variants available today, which can be utilized for high-throughput genome editing, silencing and transcriptional control with improved specificity and reduced off target effects in various systems, from yeast, *Drosophila*, bacteria, monkey, zebrafish, human, and plants.

### The Native Cas9

The DSB created by a native Cas9 can be repaired by either HR or NHEJ method (Wyman and Kanaar, 2006; Shuman and Glickman, 2007). HR-mediated repair can be used to introduce specific point mutations like nucleotide substitutions or to insert desired sequences through recombination of the target locus with exogenously supplied DNA templates (Jiang et al., 2013) (**Figure 1**). NHEJ can, however, lead to the efficient introduction of insertion/deletion mutations (*indels*), which can disrupt the translational reading frame of a coding sequence or the transcription factors binding sites in promoters or enhancers (Cong et al., 2013). The high rate of alterations after DSBs created by Cas9, makes easy identification of the desired mutations without drug-resistance marker selection (Shimatani et al., 2015). So far, the applicability of the native Cas9/sgRNA system has been demonstrated in 10 plant species including model crops for targeted mutagenesis to gene knockouts and replacement as well as multiplex plant gene editing (**Table 1**).

**Table 1:** List of applications of Cas9/sgRNA system in various plant species for single or multiplex genome editing and gene insertion or replacement.

Plant species	Target genes	Reference
**Gene knockout or editing with Cas9/sgRNA**		
*Arabidopsis thaliana*	*AtPDS3, AtFLS2, TT4,BRI1, JAZ1, GAI,ADH1, CHLI, AP1, FT, SPL4, AtCRU3, At1g56650*	Feng et al., 2013; Jiang et al., 2013; Li et al., 2013; Mao et al., 2013; Fauser et al., 2014; Feng et al., 2014; Jiang et al., 2014; Hyun et al., 2015; Johnson et al., 2015; Ma et al., 2015
*Nicotiana benthamiana*	*NbPDS, PDS, NbPDS, NbPDS3, NbIspH*	Jiang et al., 2013; Li et al., 2013; Nekrasov et al., 2013; Upadhyay et al., 2013; Yin et al., 2015
*Nicotiana tabacum*	*NtPDS, NtPDR6, ALS*	Baltes et al., 2014; Gao et al., 2014
*Oryza sativa*	*OsPDS*,*OsBADH2*,*Os02g23823*,*OsMPK2, OsSWEET11*, *OsSWEET14, CAO1, LAZY1, OsMPK5, OsMYB1, ROC5, SPP, YSA.BEL,SWEET13/1a/1b, PMS3, EPSPS, DERF1, MSH1, MYB5, CDKB2*, *OsGSTU, OsMRP15, OsAnP*, *OsAOX1a, OsAOX1b, OsAOX1c, OsBEL*	Feng et al., 2013; Jiang et al., 2013; Mao et al., 2013; Miao et al., 2013; Shan et al., 2013; Xie and Yang, 2013; Endo et al., 2014; Xu et al., 2014, 2015; Zhang et al., 2014; Zhou et al., 2014;
*Triticum Aestivum*	*TaMLO,INOX, PDS,TaMLO-A1*	Shan et al., 2013; Upadhyay et al., 2013; Wang et al., 2014
*Sorghum bicolor*	*DsRED2*	Jiang et al., 2013
*Marchantia polymorpha*	*MpARF1*	Sugano et al., 2014
*Citrus sinensis*	*CsPDS*	Jia and Wang, 2014
*Solanum lycopersicum*	*SlAGO7,mGFP5, eGFP, RIN, ANT1*	Brooks et al., 2014; Ron et al., 2014; Cermak et al., 2015; Ito et al., 2015
*Zea mays*	*ZmIPK*, *LIG1, Ms26, Ms45*, *ALS2*	Liang et al., 2014; Svitashev et al., 2015
*Glycine max*	*Glyma07g14530, Glyma06g14180, Glyma08g02290, Glyma12g37050, Glyma18g04660, Glyma20g38560; GmFEI2, GmSHR*	Cai et al., 2015; Jacobs et al., 2015; Michno et al., 2015; Sun et al., 2015
*Medicago trancatula*	*GUS*	Michno et al., 2015
*Populus tomentosa*	*PtoPDS, 4CL, PtPDS*	Fan et al., 2015; Tingting et al., 2015; Zhou et al., 2015
*Solanum tubersum*	*StIAA2*, *StALS1*	Butler et al., 2015; Wang et al., 2015
**Gene knockout or editing with Cas9 paired nickase/sgRNA**		
*Arabidopsis thaliana*	*RTEL1, ADH1, TT4*	Fauser et al., 2014; Schiml et al., 2014
**Multiplex genome editing with Cas9/sgRNA**		
*Arabidopsis thaliana*	*AtRACK1b+AtRACK1c, CHLI1+CHLI2, ETC2, CPC, TRY, and PYL1-6, At5g55580*	Li et al., 2013; Mao et al., 2013; Xing et al., 2014; Ma et al., 2015; Zhang et al., 2015
*Nicotiana tabacum*	*NtPDS+NtPDR6*	Gao et al., 2014
*Oryza sativa*	*CDKB1, CDKA1, MPK1/2/5/6, PDSOsFTL*	Endo et al., 2014; Ma et al., 2015; Xie et al., 2015
*Solanum lycopersicum*	*Solyc07g021170*+*Solyc12g044760*	Brooks et al., 2014
*Zea mays*	*ZmHKT1*	Xing et al., 2014
*Triticum aestivum*	*TaMLO-A1+TaMLO-B1+TaMLO-D1*	Wang et al., 2014
*Glycine max*	*01gDDM1*, *11gDDM1*, *01g+11gDDM1; GmFEI2, and GmSHR*	Cai et al., 2015; Jacobs et al., 2015
*Populus tomentosa*	*PtPDS1 and PtPDS2*	Tingting et al., 2015
**Gene insertion or replacement by HR with Cas9/sgRNA and donor template**		
*Arabidopsis thaliana*	*YFFP,GU.US, DGU.US → GUS functional, nptII → ADH1*	Feng et al., 2013; Mao et al., 2013; Fauser et al., 2014; Schiml et al., 2014
*Nicotiana benthamiana*	*AvrII site → NbPDS*	Li et al., 2013
*Oryza sativa*	*OsPDS*,*OsBADH2*	Shan et al., 2013
*Zea mays*	*UBI:MoPAT*	Svitashev et al., 2015


### The Cas9 Nickase

**FIGURE 2:**
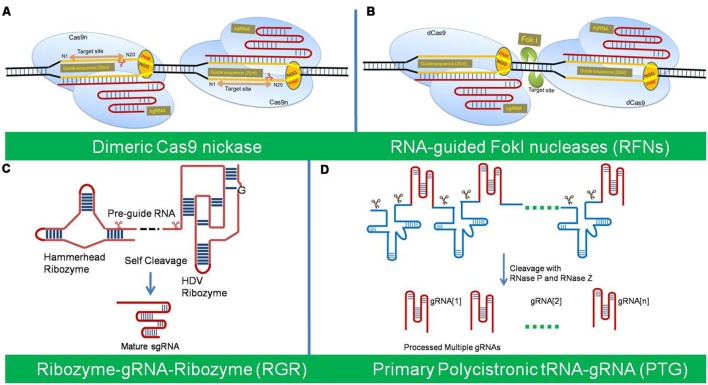
**The different variants of Cas9/sgRNA system of genome editing.** **(A)** The Cas9 nickase (Cas9n) with a RuvC or HNH mutation create a nick instead of a DSB at the target site. The dimeric Cas9n can be used for enhances specificity and reduced off target effects. **(B)** Dimeric RNA-guided FokI nucleases (RFNs) are the fusion of a catalytically inactive dCas9 protein with the FokI nuclease domain. Dimerization of two RFNs used for high genome editing frequencies and reduced off-target mutations. **(C)** Ribozyme-gRNA-Ribozyme (RGR) is an artificial gene, which generates self-catalyzed desired gRNA after transcribed from any promoter for tissue-specific genome editing. **(D)** Primary Polycistronic tRNA-gRNA (PTG) is tandemly arrayed tRNA-gRNA units, which is cleaved by the endogenous tRNA-processing system for simultaneously targeting multiple sites.

A Cas9 nickase (Cas9n) was first developed by Cong et al. (2013) through a mutation in native Cas9 (D10A, aspartate to alanine substitution). The Cas9 nickase with a RuvC or HNH mutation has the ability to create a nick, instead of a DSB at the target site. The individual nicks in the genome can be typically repaired with high fidelity homology-directed repair (HDR). The Cas9n has been used into a paired nickase system with two different gRNA to extend the number of specifically recognized bases for target cleavage, which has improved the specificity and helped mitigate the off-target phenomena (Cong et al., 2013; Mali et al., 2013; Ran et al., 2013; Fauser et al., 2014). The paired-nicking strategy has high-efficiency of HDR with reduced off-target cleavages by 50- to 1,500-fold in human cells, without sacrificing on-target cleavage efficiency (Ran et al., 2013) (**Figure 2A**).

### The Inactive dCas9

**FIGURE 3:**
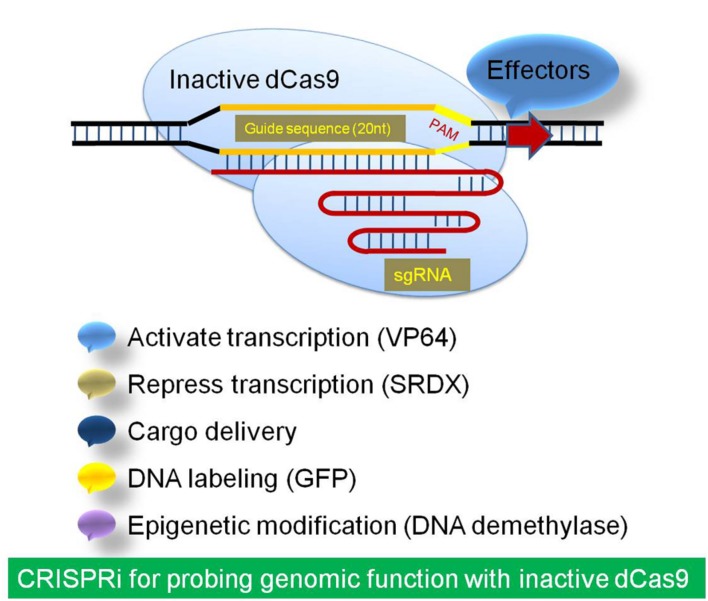
**The strategic demonstration of inactive Cas9 system for Regulation of transcription and effector delivery.** The inactive dCas9 is a catalytically inactive mutant repurposed for RNA-guided transcription regulation for high efficiency and specific CRISPR interference (CRISPRi). It has been used by fusion of dCas9 to effector domains like activator or repressor for RNA-guided DNA modulation of transcription. The dCas9 has also been used to deliver specific effectors to targeted genomic locations like GFP and DNA demethylase.

The nuclease deficient catalytically inactive mutant version of Cas9 (dCas9) has been used for RNA-guided transcription regulation, instead of genome editing (Gilbert et al., 2013; Qi et al., 2013). This modified system has been used for CRISPR interference (CRISPRi) and CRISPR activator (CRISPRa) for highly efficient and precise gene silencing and activation, respectively, using dCas9 with an effector and sgRNA. The dCas9 has an ability to incorporate gRNA and binding to the target (Xu et al., 2014). In comparison to the RNAi concept of transcript degradation and/or translation blocking, the CRISPRi system blocks the transcription initiation and elongation when dCas9/sgRNA is fused with a repressor. Therefore, dCas9/sgRNA system offers a general platform for RNA-guided DNA targeting for stable and efficient modulation of transcription. The dCas9 has been fused to effector domains with distinct regulatory functions for functional mapping of promoters and other genomic regulatory modules (Gilbert et al., 2013; Qi et al., 2013; **Figure 3**).

Lowder et al. (2015) developed a CRISPR/Cas9 toolbox of applications in plants by transcriptional regulation. They have examined the applicability of the fusing the deactivated Cas9 with the transcriptional activator VP64 or the transcriptional repressor domain SRDX for transcriptional activation and repression of multiple endogenous genes (Lowder et al., 2015). The dCas9 has also been used to deliver GFP to targeted genomic locations (Anton et al., 2014). The novel light and chemical inducible dCas9 system have been developed, which could be utilized for light and agrochemical mediated transcription activation using Cas9 system. These will further, widen the applicability of Cas9/sgRNA system in crop improvement and functional genomics (Cong et al., 2013; Polstein and Gersbach, 2015; Zetsche et al., 2015).

### Dimeric RNA-Guided FokI Nucleases (RFNs)

Dimeric RNA-guided FokI Nucleases (RFNs) are a fusion of a catalytically inactive dCas9 protein with the FokI nuclease domain. Dimerization of two RFNs rather than co-localization is required for efficient genome editing activity, which gives a plus point over the Cas9 nickase for high genome editing frequencies and reduced off-target mutations (Tsai et al., 2014; Bortesi and Fischer, 2015). Their cleavage activity depends strictly on the binding of two gRNAs with a defined spacing and orientation, which reduces the likelihood of target site occurring more than once in the genome (Tsai et al., 2014) (**Figure 2B**).

## Guide RNA Variants

There are some other modifications in the guide RNA of the Cas9/sgRNA system, which have provided other improvements in the native system for utilization in broader applicability of this significant technology.

### Truncated Guide RNAs (truRNA)

Truncated guide RNAs (truRNA) are the sgRNA variants with shorter regions of target complementarities that is 17 nucleotides in length, which offers a simple, effective strategy to improve the specificities of Cas9 nucleases or paired nickases by reducing the off-target effects (Fu et al., 2014; Bortesi and Fischer, 2015). The truncated gRNAs, with shorter regions of target complementarities can decrease undesired mutagenesis at some off-target sites without sacrificing on-target genome editing efficiencies (Fu et al., 2014).

### Ribozyme-gRNA-Ribozyme (RGR)

Ribozyme-gRNA-Ribozyme (RGR) is an artificial gene which generates RNA molecule with ribozyme sequences, which undergo self-catalyzed cleavage to generate the desired gRNA both *in vitro* and *in vivo* (Gao and Zhao, 2014b). RGR can be transcribed from any type of promoter and thus allow tissue-specific genome editing and efficient detection of mutations (**Figure 2C**).

### Polycistronic tRNA-gRNA (PTG/Cas9)

Polycistronic tRNA-gRNA (PTG/Cas9) consists of array of tandem tRNA-gRNA units, with each gRNA containing a target-specific spacer for simultaneously targeting multiple sites (Xie et al., 2015). The primary transcript of PTG is cleaved after precise processing via the endogenous tRNA-processing system by RNase P and RNase Z, which releases numerous mature gRNAs *in vivo* from a synthetic polycistronic gene. The excised mature gRNAs direct Cas9 to multiple targets which significantly increase CRISPR/Cas9 multiplex editing efficiency in plants. Xie et al. (2015) demonstrated that targeting one gene with two gRNAs using PTG would greatly increase the efficiency of complete gene knock-out in comparison to the sgRNA (**Figure 2D**).

## The Cas9/sgRNA System for Plant Genome Editing

There are broadly three categories of applications of the RNA guided endonuclease particularly in plants. First, in which DSBs created by Cas9 were repaired by non-homologous end joining (NHEJ) method for generation of *indels*, which leads to frame-shift mutations similar to natural variants, or those produced by physical or chemical mutagenesis as in mutation breeding (Chen and Gao, 2014; Saika et al., 2014). In second category, a short DNA repair template or a transgene has been used with Cas9 to repair DSB by homologous recombination (HR) for generation of the point mutations or targeted transgene insertion, gene replacement and gene stacking at predetermined sites. This avoids the position effects associated with random insertion of genes into plant genomes using genetic engineering. Third category uses, the multiplex genome editing for targeting multiple different sites with multiple sgRNAs along with the Cas9 nuclease. The multiplex genome editing in plants can be used for dissecting the functions of gene family members with redundant functions and for analyzing epistatic relationships in genetic pathways (Xing et al., 2014). Here, we have briefly reviewed the achievements of Cas9 mediated genome editing in plants (**Table 1**).

### CRISPR Achievements in Plants

The CRISPR/Cas system generates stable and heritable mutations, which can easily segregate from the Cas9/sgRNA construct to avoid further modifications by CRISPR/Cas. This results in development of homozygous modified transgene free plants in only a generation (Brooks et al., 2014; Fauser et al., 2014; Feng et al., 2014; Gao and Zhao, 2014a; Jiang et al., 2014; Schiml et al., 2014; Zhang et al., 2014; Zhou et al., 2014). Xu et al. (2015) successfully developed transgene-free rice with desired gene mutation by segregating out the transgene with self-fertilization in the T1 generation. The relative cleavage efficiency of Cas9 nucleases has been found better in comparison to previously- described TALENs and ZFNs against the same target sites (Gaj et al., 2013; Johnson et al., 2015). Xing et al. (2014) have developed a toolkit for multiplex genome editing in plants using CRISPR/Cas9-based binary vector set and a gRNA module vector set. This will facilitate transient or stable expression of CRISPR/Cas9 in a variety of plant systems and is especially useful for high-efficiency multiplex plant genome editing (Xing et al., 2014). Hence, the delivery of just two components, i.e., Cas9 and sgRNA to the host cell by genetic transformation methods is the only requirement for plant genome editing.

Baltes et al. (2014) have suggested that Gemini virus replicons (GVRs) can be used to deliver Cas9/sgRNA to plant cells with enhanced mutagenesis, in case when replication initiation protein gene (REP) was co-transformed with the Cas9/sgRNA construct. Further, to exploit the usefulness of this technology for trait discovery and development, efficient delivery methods like viruses based DNA replicons could be used for delivery of genome engineering reagents to all plant parts with higher repair frequencies and getting seeds with the desired modifications, without transformation (Baltes et al., 2014; Ali et al., 2015b). Two recent reports of direct delivery using the *Tobacco rattle virus* (TRV) (Ali et al., 2015b) and *Cabbage Leaf Curl virus* (CaLCuV) (Yin et al., 2015) have clearly demonstrated the feasibility of different virus medited Cas9/sgRNA delivery for efficient plant genome editing.

### CRISPRi in Plants

The CRISPRi (CRISPR interference) has been demonstrated for RNA-guided, stable and efficient modulation of transcription of target genes in plants by fusion of inactivated dCas9 to effector domains (Larson et al., 2013). dCas9 has been used for functional genetics for regulation of gene expression and novel synthetic biology applications. It has been recruited to specific DNA sequences by gRNAs as a fusion protein with the activation or repression domain of a transcription factor (Gilbert et al., 2013; Piatek et al., 2014). The transcription of both a reporter construct and the endogenous PDS gene in *Nicotiana benthamiana*, have been modulated by fusing the dCas9 C-terminus to the EDLL domain as transcriptional activators, and to the SRDX domain as a repressor (Piatek et al., 2014). The recognition complex of dCas9/sgRNA/effector interferes with transcriptional regulation for efficient sgRNA dependent inducible and reversible inhibition of gene expression (Qi et al., 2013; Laganà et al., 2014). Gilbert et al. (2015) identified that the target site for effective CRISPRi should lie from -50 to +300 bp relative to the Transcription Start Site (TSS) of a gene.

CRISPR activator (CRISPRa) system modulates gene expression over a ∼1,000-fold range by expression of a single sgRNA with one binding site (Gilbert et al., 2015). Development of genome-scale CRISPRi and CRISPRa libraries, would prove to be powerful tools for mapping complex stress-related signaling pathways in plants for functional genomics analysis (Laganà et al., 2014; Tingting et al., 2015; Gilbert et al., 2015). The available online resources of the CRISPR/Cas system’s materials and tools have made the wide adoption and applications of this technique very simple (**Table 2**). These include the web resources for CRISPR/Cas system and software tools for sgRNA designing with minimized off-target effects.

**Table 2:** Available online resources for CRISPR/Cas system.

Name	Remarks	Reference
Addgene	Reagents and resources	https://www.addgene.org/crispr/
sgRNA Designer	Guide RNA Design tool	http://broadinstitute.org/rnai/public/analysis-tools/sgrna-design
Cas9 Design	Guide RNA DesignTool	http://cas9.cbi.pku.edu.cn
CHOPCHOP	Target sites finding tool	https://chopchop.rc.fas.harvard.edu
CRISPR Design	Design and analysis of Guide RNA	http://crispr.mit.edu
CRISPR Genome Analyzer	Genome editing experiment analysis plateform	http://crispr-ga.net
CRISPR-PLANT	Genome-wide gRNAs prediction tool in plants	http://genome.arizona.edu/crispr
CRISPRseek	Target-specific guide RNAs design tool	http://bioconductor.org/packages/release/bioc/html/CRISPRseek.html
DNA 2.0 gRNA Design Tool	gRNA Design tool	https://dna20.com/eCommerce/cas9/input
E-CRISP	Target sites design tool	http://e-crisp-test.dkfz.de/E-CRISP
RGEN Tools	Potential off-target sites prediction tool	http://rgenome.net/cas-offinder
sgRNAcas9	sgRNA design and potential off-target sites prediction tool	http://biootools.com
CRISPR MultiTargeter	Multiplex design tool	http://multicrispr.net/
CRISPR-P	Guide RNA design in plants	http://cbi.hzau.edu.cn/crispr/
AGEseq	Analysis of Genome Editing by Sequencing	https://github.com/liangjiaoxue/AGEseq
Stupar Lab’s CRISPR Design	Target sites identifier	http://stuparcrispr.cfans.umn.edu/CRISPR/


### Virus Interference in Plants: the CRISPR Approach

The CRISPR/Cas system of genome editing has been used as a tool for imparting resistance to viruses in plants (Chaparro-Garcia et al., 2015). Three recent reports have described the CRISPR/Cas approach for protection to plants against geminiviruses (Ali et al., 2015a; Baltes et al., 2015; Ji et al., 2015). This system conferred enhanced resistance to the plants against the geminiviruses species including BCTV (Beet curly top virus), TYLCV (Tomato yellow leaf curl virus), and MeMV (Merremia mosaic virus) at the same time with single gRNA (Ali et al., 2015a); BeYDV (Bean yellow dwarf virus) (Baltes et al., 2015), and BSCTV (Beet severe curly top virus) (Ji et al., 2015).

## Regulations and Social Acceptance of CRISPR Edited Crops

**FIGURE 4:**
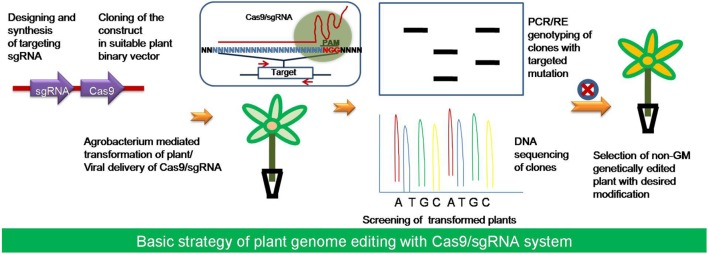
**The strategy of using plant genome editing by Cas9/sgRNA system.** Starting from the selection of the target gene, the available online resources has been utilized for designing and synthesis of sgRNA. The target sgRNA along with the suitable Cas9 variant have been cloned into a plant binary vector for transformation of the target plant species with *Agrobacterium* generally. After transformation the putative transformed plants would be selected for the presence of the Cas9 and sgRNA. Then screening of the plants with the desired mutation or editing would be done using PCR/RE genotyping and DNA sequencing.

**FIGURE 5:**
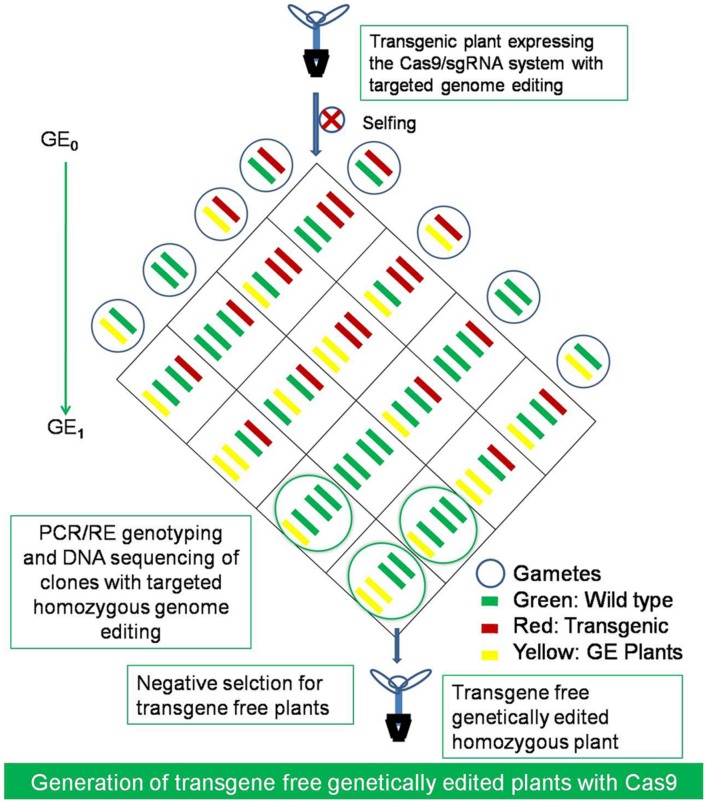
**The generation of transgene free genetically edited (GE) crops.** The transgene free homozygous mutants with desired genetic modifications at the targeted loci and without RGEN transgene construct could be selected by selfing of GE_0_ generation plants and after segregation of the transgene in the next GE_1_ generation. The GE plants could be selected by PCR/RE genotyping and DNA sequencing of clones and negatively selecting for the transgene free plants with desired modification in the first generation only.

Routine targeted mutagenesis in plants by the CRISPR/Cas9 system will definitely help open up new dimensions in plant biology research. The conventional random mutagenesis approaches were not able to access every gene for inactivation study because of the random nature of the gene integration. CRISPR-Cas9 technology would be of great help in generating mutants for inaccessible genes, mutate multiple loci and generate large deletions, which can, therefore, accelerate plant breeding without actually introducing a transgene (**Figure 4**). Although, genetically modified crops could have been a solution for crop improvement, if the controversies concerning the probable environmental and health implications of GM crops could be avoided (Hilbeck et al., 2011). Using the technique of genetic engineering a DNA construct can be directly inserted into one or more chromosomes in a random manner for manipulation of the genome. However, the random gene insertions can have undesirable effects and are not favorable for making large intensive changes, such as in case of adding an entire metabolic pathway into a plant (Lau et al., 2014). The plant breeding techniques have been used regularly to introduce new traits into cultivated varieties using existing natural genetic variation and random mutagenesis. The CRISPR/Cas system introduces precise modifications into a plant genome, which inherits stably and transgene region could be removed simply after the target gene editing to make transgene free plants during crop variety improvement (Mahfouz et al., 2014; Gao and Zhao, 2014b; Kanchiswamy et al., 2015; Xu et al., 2015; **Figure 5**). The Cas9/sgRNA system, is now one of the new plant breeding techniques (NPBT) along with the ZFNs (Zinc Finger Nucleases) and TALENs (Transcription activator like effector nucleases) like genome editing technologies. The NPBTs are faster than traditional breeding methods and can produce a null segregant line that notably lacks the transgenic insert (Podevin et al., 2012; Araki and Ishii, 2015; Schaart et al., 2015; Woo et al., 2015). The plants developed by NPBTs are identical to the classically bred plants and these should be evaluated according to the resulting end product rather than the process of creation (Giddings et al., 2012; Hartung and Schiemann, 2014; Woo et al., 2015). Thus, in such a case CRISPR edited plants might be out of the current GMO regulations, but still this pose new challenges for the regulation and social acceptance of genetically edited (GE) crops (Voytas and Gao, 2014; Araki and Ishii, 2015; Jones, 2015; Kanchiswamy et al., 2015). NPBTs are currently in debate by advisory and regulatory authorities in relation to the GMO legislation to classify GE crop varieties produced using genome editing as non-GM for the social acceptance (Lusser et al., 2012; Voytas and Gao, 2014; Araki and Ishii, 2015).

Araki and Ishii (2015) suggested a regulatory concept for GE crops based on the regulatory responses from the authorities in the world by categorizing the genome edited organisms under product- based and process-based GMO regulations. They recommend that those crops which have been engineered at gene/genome level using CRISPR-Cas9 technology should not be considered GMO under product based GMO regulation, because the final products are not genetically modified organism. While more work is needed to optimize the CRISPR/Cas system in plants as this ultimate plant genome editing toolkit shows encouraging potential for producing more complex useful agronomic traits in plant varieties (Quétier, 2016). This technology can better supplement the classical breeding techniques for understanding the complex quantitative traits, whose combinations of genes are selected by traditional breeding methods (Fichtner et al., 2014). The GE crops with the appropriate regulatory structures in place might prove to be more acceptable than the plants that carry foreign DNA in their genomes (Lozano-Juste and Cutler, 2014; Osakabe and Osakabe, 2015). The US Department of Agriculture has indicated that GE plants without any foreign DNA will not be considered as GMOs, however, the European Commission is expected to publish in the near future about the regulatory uncertainty of genome editing (Jones, 2015). The extent of the potential of CRISPR technology in applied plant research and crop breeding benefit for world’s food security will depend upon the performance and public perception of the GE crop varieties (Belhaj et al., 2015; Jones, 2015; Wolt et al., 2015).

## Future Prospects

**FIGURE 6:**
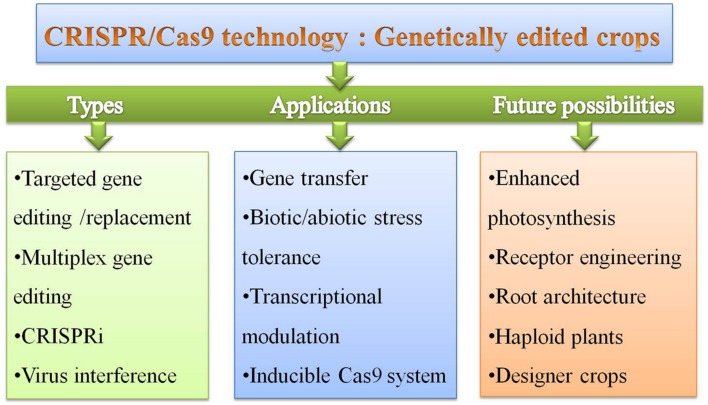
**The types, applications and future possibilities of CRISPR/Cas9 system for development of GE crops for crop improvement**.

The potential future crops for sustainable productive agriculture by genome editing are those which have better pest resistance, with enhanced nutritional value, and that are able to survive in changing climate. Climate resilient agriculture for combating abiotic and biotic stress is the future of crop improvement using genome editing for both the targeted mutagenesis mediated manipulation and study of transcriptional control by dissection of physiological and molecular cross talk under combined stress (Kissoudis et al., 2014; Jain, 2015). Genome editing will play very important role in developing new bio-energy crops, which could give maximum yield on wastelands and changing climate (Bosch and Hazen, 2013). This technology could offer any possible novel genome-editing concept for plants in order to improve crops for better nutrition and food security. We here suggest some of the possible concepts, which could be utilized for crop improvement and plant biotechnology applications (Liu and Fan, 2014) (**Figure 6**). Further, direct delivery methods of Cas9 and gRNA using *Agrobacterium* and Viral replicons by using nanoparticles can be very useful for simplifying the genome editing technology (Hiei et al., 2014; Khatodia et al., 2014; Nonaka and Ezura, 2014). Inducible Cas9 system for transcription modulation like split-Cas9 for chemically inducible system and light activated Cas9 effector (LACE) could be utilized for crop improvement in future (Polstein and Gersbach, 2015; Zetsche et al., 2015). The generation of large-scale whole-genome targeted sgRNA library for high-throughput loss-of-function screening applications based on the CRISPRi system like that of RNAi is particularly feasible for model plants in future (Heintze et al., 2013).

Root trait is very important target for crop improvement using the CRISPR/Cas9 genome editing by studying the regulation of stress responses at cellular level in roots, allele replacement for QTL validation, and the epigenetic regulation of roots (Ahmadi et al., 2014). A major breeding target using CRISPR/Cas9 genome editing is to build cereal crops harboring root systems that can capture unevenly distributed water and nutrient resources in climate instability and resource scarcity. Targeted mutagenesis using the CRISPR-Cas9 system can bring advances in functional genomic research of legumes especially by generating the target mutants of the genes involved in roots and nodules (Sun et al., 2015). A recently published report of naturally transgenic sweet potato having T-DNAs has raised a few questions (Kyndt et al., 2015). The CRISPR/Cas9 editing methods can be used to delete T-DNAs for ‘non-transgenic’ sweet potato generation to test whether the T-DNAs confer a useful phenotype in naturally transgenic sweet potato (Jones, 2015). This technology can also be utilized for the production of haploid plants by alterations in the histone proteins found in centromeric nucleosomes (Kumar and Jain, 2014). Centromeric histone H3 protein (CENH3) is a member of the kinetochore complex, which is required for kinetochore formation and for chromosome segregation. Using the CRISPR/Cas9 technology, the expression of endogenous CENH3 gene can be suppressed by targeted mutagenesis in a plant, which can lead to selective loss of one set of chromosomes for haploid plants production (Maheswari et al., 2014). The ease and multiplicity of CRISPR/Cas system have shown the potential in three dimensions of plant functional genomics, i.e., genomics, transcriptomics, and epigenomics (Puchta, 2016). This can allow simultaneous induction as well as repression of certain sets of genes and at the same time aid in reprogramming the epigenome (Puchta, 2016).

In conclusion, this technique is getting more precise and efficient day by day, as the future opportunities like inducible Cas9 expression and direct delivery of Cas9 protein, are being explored in different organisms and cell types among the biologists all over the world (Ramakrishna et al., 2014; Polstein and Gersbach, 2015). These novel improvements can help avoid the boosting of off-target effects by expressing the Cas9/sgRNA only when required. Plant biotechnological applications of Cas9 technology not only lies in engineering non-food crops but also have the potential of generating altogether new plant varieties/species for production of specialized chemicals and biomaterials. The swiftness at which Cas9/sgRNA system has developed and improved, it has actually provided human control over genetic information with a huge genomic revolution. This will revolutionize both basic and applied research to improve a wide variety of agronomic traits in crop plants. Another remarkable worth of this CRISPR/cas9 system is that it is by far the most user friendly technique among all the currently available genome editing techniques.

## Author Contributions

SK and KB conceived and wrote the manuscript. NP reviewed and helped in figures. SMPK and NT reviewed the manuscript. All authors listed, have made substantial, direct, and intellectual contribution to the work, and approved it for publication.

## Conflict of Interest Statement

The authors declare that the research was conducted in the absence of any commercial or financial relationships that could be construed as a potential conflict of interest.
